# A novel Bayesian approach to quantify clinical variables and to determine their spectroscopic counterparts in ^1^H NMR metabonomic data

**DOI:** 10.1186/1471-2105-8-S2-S8

**Published:** 2007-05-03

**Authors:** Aki Vehtari, Ville-Petteri Mäkinen, Pasi Soininen, Petri Ingman, Sanna M Mäkelä, Markku J Savolainen, Minna L Hannuksela, Kimmo Kaski, Mika Ala-Korpela

**Affiliations:** 1 Laboratory of Computational Engineering, Systems Biology and Bioinformation Technology, Helsinki University of Technology, P.O. Box 9203, FI-02015 HUT, Finland; 2 Department of Chemistry, University of Kuopio, P.O. Box 1627, FI-70211 Kuopio, Finland; 3 Department of Chemistry, Instrument Centre, Vatselankatu 2, FI-20014 University of Turku, Turku, Finland; 4 Department of Internal Medicine, Clinical Research Center, University of Oulu, P.O. Box 5000, FI-90014 Oulu, Finland

## Abstract

**Background:**

A key challenge in metabonomics is to uncover quantitative associations between multidimensional spectroscopic data and biochemical measures used for disease risk assessment and diagnostics. Here we focus on clinically relevant estimation of lipoprotein lipids by ^1^H NMR spectroscopy of serum.

**Results:**

A Bayesian methodology, with a biochemical motivation, is presented for a real ^1^H NMR metabonomics data set of 75 serum samples. Lipoprotein lipid concentrations were independently obtained for these samples via ultracentrifugation and specific biochemical assays. The Bayesian models were constructed by Markov chain Monte Carlo (MCMC) and they showed remarkably good quantitative performance, the predictive R-values being 0.985 for the very low density lipoprotein triglycerides (VLDL-TG), 0.787 for the intermediate, 0.943 for the low, and 0.933 for the high density lipoprotein cholesterol (IDL-C, LDL-C and HDL-C, respectively). The modelling produced a kernel-based reformulation of the data, the parameters of which coincided with the well-known biochemical characteristics of the ^1^H NMR spectra; particularly for VLDL-TG and HDL-C the Bayesian methodology was able to clearly identify the most characteristic resonances within the heavily overlapping information in the spectra. For IDL-C and LDL-C the resulting model kernels were more complex than those for VLDL-TG and HDL-C, probably reflecting the severe overlap of the IDL and LDL resonances in the ^1^H NMR spectra.

**Conclusion:**

The systematic use of Bayesian MCMC analysis is computationally demanding. Nevertheless, the combination of high-quality quantification and the biochemical rationale of the resulting models is expected to be useful in the field of metabonomics.

## Background

Genomics is increasingly complemented by *metabonomics *– the quantitative measurement of the time-related multiparametric metabolic responses of multicellular systems to (patho)physiological stimuli or genetic modification [1]. Mass spectrometry and nuclear magnetic resonance (NMR) spectroscopy have become the two key technologies in the metabonomic field [2]. An appealing feature of NMR spectroscopy for metabonomic applications is its specific yet non-selective nature: proton (^1^H) NMR can efficiently produce information on a large number of metabolites in biological samples like human serum. The abundance of protons and the inherently narrow as well as heterogeneous chemical shift range of ^1^H NMR results in highly informative spectra that contain heavily overlapping resonances [3].

Recently, a call for applying ^1^H NMR metabonomics to facilitate disease risk assessment and clinical diagnostics has emerged [1,2,4-8]. A key issue in bringing metabonomics for clinical use will be to bridge the gap between biochemistry – as revealed by ^1^H NMR spectroscopy – and the relevant measures of current clinical practice. In a ^1^H NMR spectrum, one metabolite can manifest several peaks, and the spectral intensities are both biochemically and (patho)physiologically related. Furthermore, the data sets are extensive but redundant: one measurement can yield tens of thousands of data points, but the effective dimensionality is much less due to a smaller number of NMR-visible compounds. Consequently, there are methodological challenges in trying to quantitatively associate ^1^H NMR metabonomics data to relevant biochemical variables as well as to understand and visualise the underlying metabolic features that relate to various biomedical applications [8].

A key clinical application of ^1^H NMR spectroscopy is to quantify lipoprotein lipids directly from plasma or serum samples [3,7,9-13]. One of the strategic reasons to use ^1^H NMR to study lipoproteins is the avoidance of their tedious physical isolation from plasma via repetitive ultracentrifugations and thus the consequent potential to analyse extensive clinical data sets beyond current biochemical methodologies. Various ^1^H NMR spectroscopy applications have focused on the main lipoprotein fractions, namely very low, intermediate, low and high density lipoproteins (VLDL, IDL, LDL and HDL, respectively), since these relate to general clinical guidelines to assess an individual's risk for atherosclerosis [3,6,12]. Interestingly, one of the advanced methods, already in clinical use, to determine plasma lipoproteins is a commercial ^1^H NMR based assay named NMR LipoProfile^® ^by LipoScience Inc [13]. Thus, ^1^H NMR spectroscopy and metabonomics of serum provides an extensively studied and demonstrative case of complex overlapping resonances with well-known biochemical rationale and spectral characteristics [3,6,7,9-13].

Biomedical research relies heavily on the statistical analysis of empirical findings and extrapolation from limited sample sets to larger populations. Currently, hypothesis testing with pre-selected parametric formulations is the prevailing technique and statistical uncertainty is expressed indirectly by comparing the observations to a given null hypothesis. In multi-dimensional applications such as ^1^H NMR metabonomics the null hypothesis is obtainable only for the simplest formulations, which are often inadequate to describe the data efficiently. In contrast, Bayesian theory [14,15] explicitly incorporates uncertainty in the form of probability distributions, hence the null hypothesis is no longer required as the reference point. Furthermore, the parametric formulations need not be pre-selected heuristically, but can be included in the modelling process itself. Hence, the analysis becomes more dependent on the data and prior knowledge, and less dependent on arbitrary practical restrictions such as analytical tractability. However, applications of Bayesian methodology in NMR spectroscopy are sparse [16-18], perhaps due to the lack of computing power until recent years. A Bayesian spectral decomposition has produced promising results for metabonomic NMR data [19] but, to our knowledge, this is the first biomedical application of Bayesian inference on spectral quantification with special modelling emphasis on the metabolic rationale.

Thus, this work has two key objectives to establish. First, to quantify broad overlapping resonances from ^1^H NMR spectra of serum using specific Bayesian models, and, second, to relate the resulting model kernels to the known biochemical characteristics of the spectra. This study focuses on a clinically significant application of ^1^H NMR spectroscopy of serum for quantifying lipoprotein lipid concentrations used for the assessment of individuals' risk for coronary heart disease. A set of biochemically characterised serum samples, for which VLDL and IDL triglycerides (VLDL-TG and IDL-TG, respectively) as well as IDL, LDL and HDL cholesterol (IDL-C, LDL-C and HDL-C, respectively) concentrations are independently measured, is the origin for the ^1^H NMR spectra. A Markov chain Monte Carlo (MCMC) in Bayesian inference is used to set up quantitative models based on these ^1^H NMR spectra and to automatically define the number and locations of Gaussian kernels to indicate the spectral features corresponding to each biochemical variable.

## Methods

### Serum samples and biochemical lipoprotein lipid analysis

The serum samples and the biochemical lipoprotein lipid analyses were available from 75 individuals, representing a wide range of plasma lipoprotein lipid concentrations. The blood samples were drawn after an overnight fast of 12 hours into EDTA-containing tubes or tubes without anticoagulant for serum separation. Blood samples for serum separation were incubated at room temperature for 30 min prior to centrifugation. Serum and plasma were separated by centrifugation at 1200 g–1500 g for 10–15 min at 4°C. The main lipoprotein fractions were isolated from plasma by sequential ultracentrifugation using density ranges of ≤ 1.006 g/ml, 1.006–1.019 g/ml, 1.019–1.063 g/ml, and 1.063–1.210 g/ml for VLDL, IDL, LDL, and HDL, respectively [20]. Cholesterol and triglyceride concentrations in lipoproteins were determined with Specific Chemistry Analyser (Kone, Finland) using enzymatic colorimetric methods (kits by Boehringer Diagnostica, Mannheim GmbH, FRG) and expressed as mmol/l plasma.

### ^1^H NMR spectroscopy

The ^1^H NMR data were recorded at the physiological temperature of 310 K on a Bruker AVANCE spectrometer operating at 500.13 MHz equipped with a 5 mm BBI probehead. A double tube system facilitating absolute metabolite quantification was used [8,10]. The external reference tube (o.d. 2 mm, supported by a Teflon adapter) containing the reference substance (sodium 3-trimethylsilyl[2,2,3,3-*d*_4_]propionate (TSP) 40 mmol/l, MnSO_4 _0.6 mmol/l in 99.8% D_2_O) was placed coaxially into the NMR sample tube (o.d. 5 mm) containing 430 μl of each sample. No water suppression was used and 128 transients were collected with a 90 degree flip angle using a spectral width of 5252 Hz and 64 k data points. Acquisition time of 6.2 s and a relaxation delay of 0.1 s were used. Prior to Fourier transformation, the measured free induction decays were zero filled and multiplied by an exponential window function with a line-broadening of 1.0 Hz. The PERCH NMR software was used for pre-processing the data [21]. The metabolite intensities in each spectrum were scaled according to the corresponding TSP reference signal before the Bayesian analyses.

### Bayesian spectral analysis

The aliphatic regions of the ^1^H NMR spectra (from 0.40 to 3.30 ppm; 18 093 data points) were analysed from the serum samples of those individuals that had the lipoprotein lipid concentrations for VLDL-TG, IDL-TG, IDL-C, LDL-C and HDL-C available. The biochemical assays for these lipid variables and the ^1^H NMR spectra are physically independent. Thus, by modelling the quantitative relation between the ^1^H NMR metabonomics data and the clinical variables, the concentrations of these lipid fractions can be estimated from the serum spectra alone. A separate Bayesian model was constructed for VLDL-TG, IDL-TG, IDL-C, LDL-C and HDL-C.

One could assume that all the data points are independent, but clearly this is not true just by looking at the smooth spectral curves. In addition, such assumption would lead to unnecessary methodological problems [14]. Here, the spectroscopic fact that adjacent data points are strongly correlated is not ignored but an unknown and non-constant correlation length is allowed. This is achieved by representing adjacent points collectively through a Gaussian kernel with a given width and location. Specifically, the dot product between the spectral intensity vector of a sample and the Gaussian density function (truncated at 3σ) represents the value of the corresponding kernel. The minimum width was constrained to fulfil the known molecular characteristics in the NMR spectra [3,8], that is, kernel widths larger than 4 Hz were favoured.

Based on the application specific knowledge, it was reasonable to assume that a linear model of the target variables and kernel outputs was appropriate [7,10]. This does not imply a fully linear model, since the mapping from the raw spectra to the kernel space is non-linear, especially since the kernel number is among the targets of the inference. In addition to the kernels, the mean level of each spectrum was used as a covariate. Student's t-distribution was preferred over the Gaussian distribution as a more robust residual model and the posterior inference was made by Markov chain Monte Carlo (MCMC) [14]. A useful property of our model specification is that marginal likelihoods, obtained by analytically integrating over the linear model weights, can be used to significantly improve the sampling quality [15]. Kernel locations and widths, and the degree of freedom for the residual model were inferred by slice sampling. In addition, the number of kernels was sampled by reversible jump MCMC in which the proposal distributions for new parameters were the corresponding prior distributions. The rest of the model parameters were updated using Gibbs' sampling with conditional distributions [14]. Interestingly, by allowing the selection of input variables to be among the targets of modelling, the effect of prior assumptions can be reduced if compared to conventional statistics. A Bayesian rationale and a brief mathematical formulation of the Bayesian modelling for the ^1^H NMR spectra of serum are given in Additional file 1.

An intuitive and practical consideration of the Bayesian methodology used here is as follows. First, the kernel outputs are computed, as specified by the locations and widths, *i.e.*, the dot products of every kernel vector and every spectrum are computed. This generates a new *n *× *k *input matrix ***ϕ***, where *n *is the number of spectra and *k *the number of kernels. These kernel features can now be connected to the target variable **y **through the (simplified) linear regression equation **y **= **w*ϕ ***+ **ε**, where **w **is the weight vector and **ε **represents the noise. To incorporate the uncertainties to the model, a sample of **w **is drawn according to the analytical posterior distribution (given current ***ϕ ***and noise distribution) instead of finding the algebraically optimal weight vector. Next, the shape parameters of the noise distribution are sampled in a similar fashion, given the current **w **and ***ϕ***. Finally, the number of kernels *k *is changed (given current **w**, ***ϕ ***and noise distribution) to try if another number could produce better results. The above cycle is repeated until convergence seems stable and enough samples of the parameters have been obtained to construct histograms that serve as approximations of the posterior parameter distributions. Note that this is only a simplified account of the algorithm used (see Additional file 1 for a more detailed methodology).

In general, the distributions of all parameters converged fast and mixed well, although the number of kernels turned out to be somewhat slow in mixing. For a single MCMC run, reliable results are obtainable at 10000 iterations or one hour on a 1 GHz Alpha EV6.8CB processor. Before inference, the first 2000 iterations were discarded and only every 20th of the rest were included. Convergence was verified by comparing 10 independent chains. Replicates from the predictive distribution of the model were computed to serve as a test set in estimating the predictive performance. In a preliminary phase, a 10-fold cross-validation was used as a more robust strategy to check that the predictive replicate approach produced meaningful results. After the posterior distribution has been constructed, predictions for new spectra can be computed almost instantaneously.

The computations were performed using the MCMCstuff toolbox [22], which is a collection of Matlab functions for Bayesian inference by Markov chain Monte Carlo methods.

## Results and discussion

### Quantitative models

**Figure 1:**
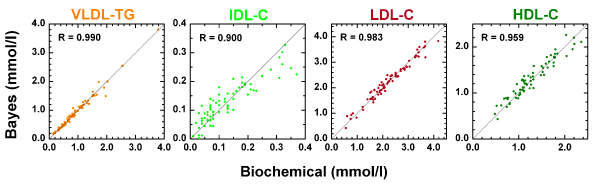
**The quantitative performance of the Bayesian models**. The results of the Bayesian models for the VLDL-TG (n = 75) (*orange*), IDL-C (n = 72) (*lime*), LDL-C (n = 72) (*sherry*) and HDL-C (n = 67) (*olive*). The correlation coefficients (R) shown are between predictions and observations (for predictive R-values see Results and discussion). The straight black lines show a 1:1-relationship and are drawn only to guide the eye.

Figure 1 shows the results of the separate quantitative Bayesian models for the VLDL-TG, IDL-C, LDL-C and HDL-C. In Figure 1 correlation coefficients (R) between predictions and observations are shown to allow consistent comparison to previous studies ([3,12] and refs. therein). Since the same observations are used to update the posterior and to compute R, these correlation values are optimistic with regard to the predictive performance of the models for future data. Thus, we also estimated the predictive correlation coefficients (predictive R) by integrating over the uncertainty related to future observations; these predictive R-values then describe how well the models can predict the corresponding lipoprotein concentrations in the case of ^1^H NMR spectra of new individuals. The predictive R-values/R-values are 0.985/0.990 (n = 75), 0.787/0.900 (n = 72), 0.943/0.983 (n = 72) and 0.933/0.959 (n = 67) for the VLDL-TG, IDL-C, LDL-C and HDL-C, respectively. It is notable that the predictive R calculated for IDL-C clearly reflects the rather large uncertainties related to the experimental determinations of low concentrations. In general, these values represent excellent quantitative correspondence in this extensively studied complex application [3,6,7,9-13]. Particularly, in the case of IDL-C and HDL-C the current results appear slightly better than those previously reported (see [12] for a comparison for the quantitative performance of different methods used). Thus, these results verify the high-quality quantification ability of the presented Bayesian MCMC approach in the case of broad overlapping resonances in the ^1^H NMR spectra of serum. This is an important prerequisite to facilitate the assessment of the biochemical rationale of the Gaussian model kernels in relation to each lipoprotein fraction and the ^1^H NMR spectroscopic characteristics of serum. The general quantification aspects of lipoprotein lipids using ^1^H NMR spectroscopy of serum (or plasma) have been extensively handled in the literature [[3,6,7,9-13] and refs. therein] and will not be discussed in detail here.

We also tried to set up an equivalent Bayesian model to quantify IDL-TG. However, a properly quantitative model was not achieved (a predictive R-value of only 0.608, n = 72; data not shown). Concerning quantification of IDL-TG and IDL-C somewhat varying results have been published [7,9,12,13]. The IDL fraction is included in the recently improved NMR LipoProfile^® ^method [23] and previously the application of neural network analysis have resulted in reasonable (semi)quantitative models [9,12]. It is also notable that all the IDL-C and IDL-TG concentrations for the current sample set are below 0.4 mmol/l. Thus, a likely reason to affect the modelling is the fact that biochemically measured concentrations below 0.5 mmol/l can contain experimental inaccuracies of several tens of percent [12]. Why the quantitative Bayesian model here for IDL-C appears rather good but the model for IDL-TG leads to more inaccuracies is not currently clear. Nevertheless, the resulting quantitative model for IDL-C can be used here to assess how the Gaussian model kernels relate to the known ^1^H NMR spectroscopic characteristics of the IDL particles.

**Figure 2:**
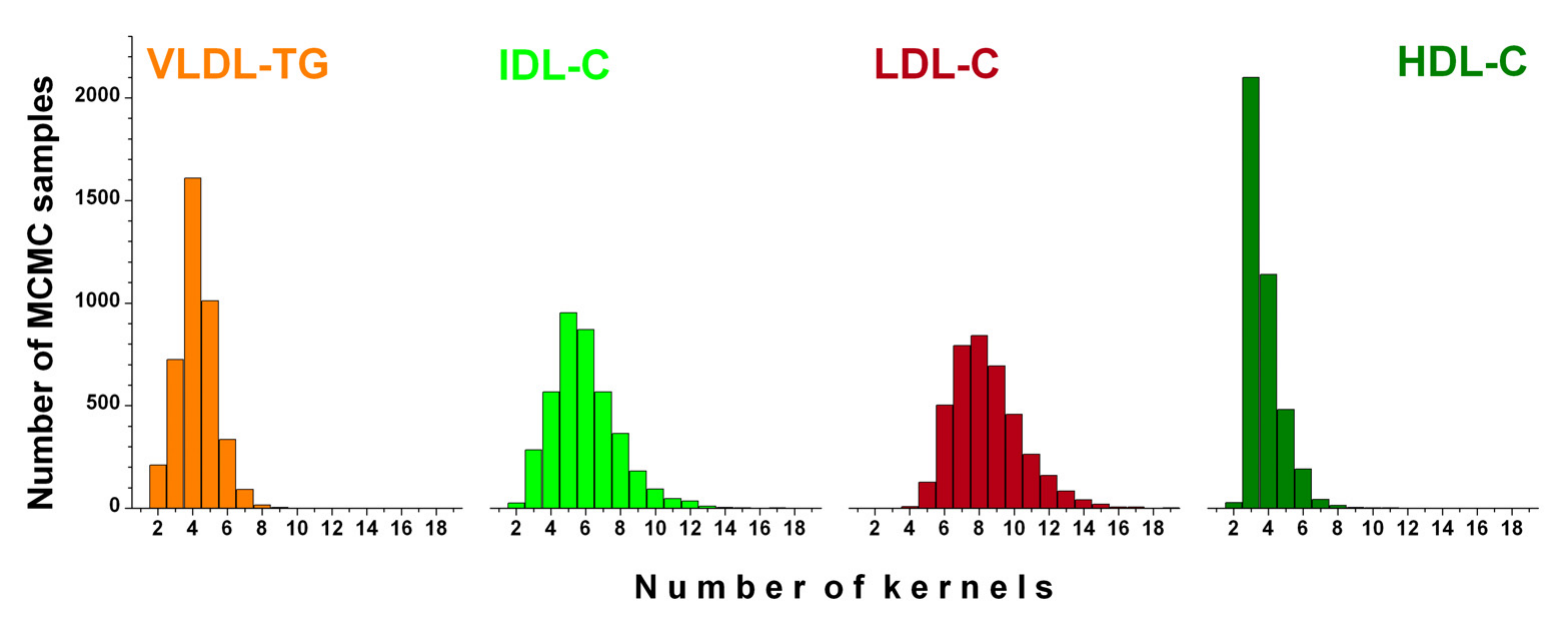
**The kernel distributions in the Bayesian models**. The marginal posterior distribution for the number of kernels in the Bayesian models for the VLDL-TG (*orange*), IDL-C (*lime*), LDL-C (*sherry*) and HDL-C (*olive*).

**Figure 3:**
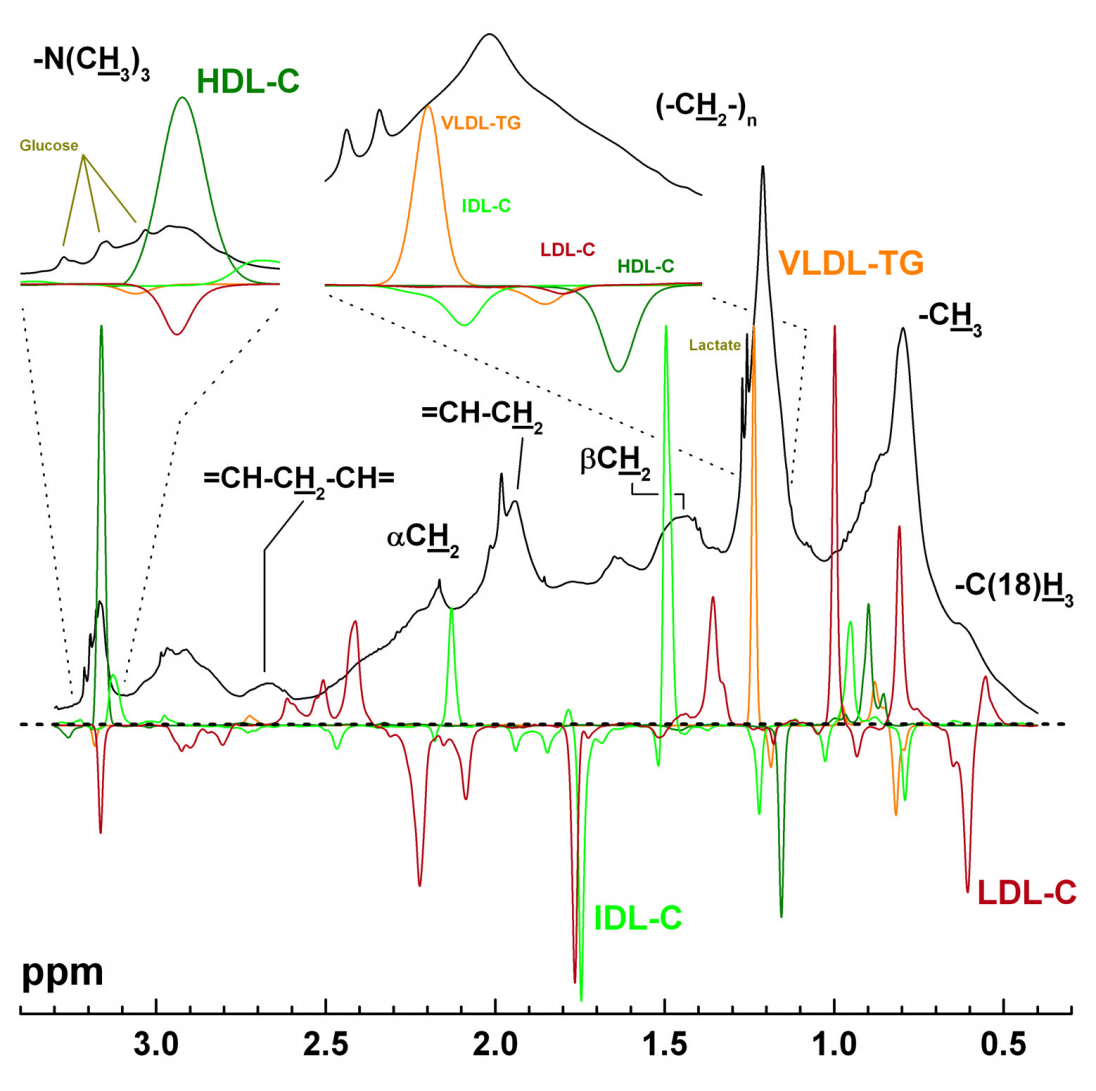
**A representative ^1^H NMR spectrum of serum and the spectroscopic characteristics of the Bayesian model kernels**. Illustration of the aliphatic spectral region of a representative experimental ^1^H NMR spectrum (*black*) together with the main Bayesian model kernels for the VLDL-TG (*orange*), IDL-C (*lime*), LDL-C (*sherry*) and HDL-C (*olive*). The assignments for the resonances refer to fatty acids in triglycerides, cholesterol compounds and phospholipids in various lipoprotein particles, the cholesterol backbone -C(18)H_3 _and the -N(CH_3_)_3 _groups of surface phospholipids. Thus, it should be noted that all the lipoprotein fractions present in serum contribute to all of these resonances. The insets show the choline -N(CH_3_)_3 _region and the lipid (-CH_2_-)_n _region in mode detail. The highest intensity kernel for each lipoprotein fraction was scaled to 1.0. The dotted horizontal line shows the zero level.

Since kernel selection was part of our modelling, it is possible to estimate the marginal distributions for the number of kernels, as depicted in Figure 2. Before any interpretation, though, the coefficient prior of the linear regression model that connects the kernel outputs and the target variable has an effect on kernel number. Fortunately the predictions are not sensitive to this since the kernel number is integrated out, but the phenomenon may discriminate models with only a few kernels and large coefficients. In any case, IDL-C and LDL-C are clearly more dispersed than VLDL-TG and HDL-C and this also translates to Figure 3, where both VLDL-TG and HDL-C are dominated by a few kernels, but IDL-C and LDL-C produce clear associations at numerous locations. Note, however, that for a pair of highly correlated but non-adjacent spectral regions you might get two strong associations, but during the MCMC simulation you might get only one of them at a time. In this respect, the number of kernels provides an additional insight to the nature of the multi-variate dependencies within the kernels.

### The biochemical rationale of the Bayesian model kernels

Typical aliphatic resonances in an experimental ^1^H NMR spectrum of human serum are illustrated in Figure 3. The characteristic spectral features include broad overlapping resonances originating mainly from different lipid molecules in lipoprotein particles, for example, the -CH_3 _groups of triglycerides, cholesterol compounds and phospholipids at around 0.80 ppm and the surface phospholipid -N(CH_3_)_3 _groups at around 3.18 ppm. Also resonances from glucose and some low-molecular-weight metabolites, such as lactate, are clearly visible in the spectrum. The broad underlying hump in the aliphatic spectral region is arising mostly from serum albumin and the albumin bounds fatty acids [3]. A fundamental aspect to keep in mind here is that all the lipoprotein fractions present in serum contribute to all of the lipid resonances (*cf.*, Figure 3). It is also known that the chemical shifts of the lipid resonances are size-dependent [3,7,24], *i.e.*, the low frequency sides of the lipid resonances represent the smaller HDL particles and the high frequency sides the larger VLDL particles. Thus, the contributions from the intermediately sized IDL and LDL particles are situated in the middle regions of the lipid resonances. The compositional differences between the different lipoprotein fractions are also known to cause some characteristic features for the ^1^H NMR spectra. These include the distinct resonance of VLDL-TG in the high frequency side of the (-CH_2_-)_n _resonance at around 1.2 ppm [3,9] and the pronounced contribution of the cholesterol compounds in LDL particles for the -CH_3 _and -C(18)H_3 _resonances at around 0.8 ppm and 0.6 ppm, respectively [3,9,13]. Also, the prominent contribution of HDL particles for the -N(CH_3_)_3 _resonance at around 3.18 ppm has recently been highlighted [7].

The resulting Gaussian kernel models for each lipoprotein fraction are illustrated in Figure 3 with colour coding: orange for VLDL-TG, lime for IDL-C, sherry for LDL-C and olive for HDL-C. The highest intensity kernel for each lipoprotein fraction is scaled to 1.0. It is evident from Figure 3, that the most influential kernel for both VLDL-TG and HDL-C is located exactly at the frequency position expected by the aforementioned well-known biochemical background and characteristics of the ^1^H NMR spectra of serum. Thus, the high frequency side of the (-CH_2_-)_n _resonance at around 1.2 ppm and the majority of the choline -N(CH_3_)_3 _resonance at around 3.18 ppm seem to be the most important locations for the quantitative Bayesian models of VLDL-TG and HDL-C, respectively. Though the contributions of the other kernels for VLDG-TG and HDL-C are far less pronounced they also match the spectroscopic characteristics remarkably well. In the case of VLDL-TG there is a tiny contribution from the high frequency side of the choline resonance and the small negative kernels within the -CH_3 _resonance region are also correctly situated at the high frequency side of the resonance. For HDL-C a clear kernel appears at the low frequency side of the (-CH_2_-)_n _resonance. The two small kernels close to 0.9 ppm are likely to relate to resonances from cholesterol compounds, known to be fairly pronounced especially in the case of HDL_2 _particles [7]. Consequently, the biochemical rationales as indicated by the resulting Gaussian kernel models for VLDL-TG and HDL-C are fully coherent with the known characteristics of the ^1^H NMR spectra of serum.

The kernel models for VLDL-TG and HDL-C contain fewer kernels and are much simpler than the corresponding models for IDL-C and LDL-C. This result and these differences are likely to represent the overlap of the lipoprotein resonances in the ^1^H NMR spectra. The molecular signals arising from the VLDL and HDL particles situate at the high and low frequency sides of the lipid resonances, respectively, while the contributions from the IDL and LDL particles are in the middle of the corresponding resonances [3,9,13]. This seems to have a marked effect on the Gaussian kernel models. Some of the individual kernels can be interpreted on the basis of the known characteristics of the ^1^H NMR spectra, for instance, the influential kernels for LDL-C at 0.6 ppm and at 0.8 ppm (*cf.*, the discussion above) and the expected frequency positioning of all the lipoprotein kernels within the (-CH_2_-)_n _resonance region as pointed up in the inset for Figure 3. Conversely, there are also several kernels that cannot be justified as clearly as those. The situation is similar in the case of the kernels for IDL-C and LDL-C. In general, the spectroscopic and biochemical aspects of the kernels for all the above discussed lipid concentrations also compare well with results from a previous approach in which neural network weights for different spectral points for different quantitative lipoprotein lipid models were assessed [9].

The quantification accuracy achieved via the Bayesian modelling is excellent also for LDL-C and good for IDL-C. The resulting more complex kernel models for IDL-C and LDL-C than for VLDL-TG and HDL-C are thus likely to represent the more severe signal overlap for IDL-C and LDL-C than for VLDL-TG and HDL-C. Since clear resonances identifiable to IDL-C or LDL-C are non-resolvable in the ^1^H NMR spectra of serum, the Bayesian logic seems to give rise to quite balanced combinations of several kernels at frequency locations where some information on the modelled biochemical measure is (known to be) available. In the case of severe signal overlap, however, the optimisation of the quantification and the resulting kernel models seems to take place at the expense of the biochemical interpretability.

The analysis of ^1^H NMR spectra seems to benefit from several characteristics of the Bayesian approach. First, feature extraction and selection from the high-dimensional raw data can be included as parts of the model. Second, any prior knowledge about the parameters can be explicitly incorporated into the framework. Third, no null hypothesis needs to be constructed. Additionally, in contrast to standard computational methods used in the area of NMR metabonomics [8], the Bayesian approach produces results that are tightly connected to the statistics and yet rather easy to interpret biochemically.

## Conclusion

A set of ^1^H NMR spectra of serum samples, for which clinically relevant lipoprotein lipid concentrations were biochemically characterised, were analysed using an automated MCMC Bayesian inference. This real metabonomic case of ^1^H NMR spectroscopy to quantify lipoprotein lipids directly from serum represents a biomedically relevant application with a well-known biochemical background and spectroscopic characteristics. To the best of our knowledge this is the first Bayesian application to quantify biomedical ^1^H NMR spectra and to relate the resulting model kernels to the known biochemical characteristics of the spectra. The results illustrate a high-quality quantification ability of the presented Bayesian MCMC approach in the case of broad overlapping ^1^H NMR resonances. If the signal overlap is severe, the resulting kernel models seem to form at the cost of the biochemical justification. In the case of more clearly resolvable resonances, the biochemical rationale of the uncomplicated kernel models appeared fully consistent with the known spectroscopic characteristics of the application. Hence, even though the Bayesian MCMC analysis is computationally demanding, it is anticipated to provide an advantageous complement to the currently used data analysis methods in the NMR metabonomics arena, not only in quantitative modelling but also in revealing metabolic rationale of the models and biomedical applications.

## Authors' contributions

AV, VPM, KK and MAK conceived and designed the study; SMM, MJS and MLH collected the serum samples, isolated the lipoprotein fractions and measured the biochemical data; PS and PI measured the NMR data; AV wrote the computer code and performed the Bayesian analyses; AV, VPM and MAK wrote the manuscript. All authors contributed to, read and approved the final manuscript.

## Supplementary Material

Additional File 1 **The Bayesian rationale and mathematical formulation**. The Bayesian rationale and a brief mathematical formulation of the quantitative Bayesian modelling for the ^1^H NMR spectra of serum. Click here for file
