# Supplementary material for: A novel Bayesian approach to quantify clinical variables and to determine their spectroscopic counterparts in 1H NMR metabonomic data
Source: BMC Bioinformatics. 2007 May 3;8(Suppl 2):S8. doi: 10.1186/1471-2105-8-S2-S8 (PMC1892077; doi:10.1186/1471-2105-8-S2-S8)
Supplement: Additional File 1 — The Bayesian rationale and mathematical formulation. The Bayesian rationale and a brief mathematical formulation of the quantitative Bayesian modelling for the 1H NMR spectra of serum. [file 1471-2105-8-S2-S8-S1.pdf]

Appendix: Bayesian rationale and mathematical formulations

Uncertainty is unavoidable in any statistical modelling due to finite sets of samples and random noise in measurements. Bayesian probability theory offers an elegant and consistent way to handle this lack of absolute confidence by representing the experimenter's uncertainty explicitly in the form of probability distributions. The inference process begins at the description of what would be observed if the data were generated by the model at hand, that is, the likelihood of data is computed given a certain model with given parameters. Since we are really interested in the parameter values, the Bayes' rule is then used to express the probability distributions of the parameters after combining the data with any prior beliefs of what the parameters might be. Note that the prior knowledge is expressed by a distribution, hence the posterior beliefs of the parameter values are communicated by another probability distribution, in contrast to the point estimates that are necessary in hypothesis testing.

Marginalisation of posterior distributions is another benefit of the Bayesian approach. The uncertainty about the unknown parameters is usually expressed by joint probabilities, but by integrating the less relevant parameters away, attention can be focused on the interesting aspects of the model. From a technical point of view, closed-form integrals are seldom possible for complex models and various approximation schemes must be employed. A popular choice is to employ Markov chain Monte Carlo (MCMC) methods to obtain samples from the posterior distribution and to use these samples in estimating marginal probabilities.

The mathematical model definition consists of several distinct modules, all con-

nected through the Bayes' rule. Suppose $X_{(i)}$ is a vector of the spectral intensities measured for the individual i. Similarly, y_i denotes the non-spectroscopic measurement that is the target of modelling with respect to the spectrum $X_{(i)}$. Based on existing spectroscopic knowledge, we assume that the information in X on the target variable y is concentrated on an unknown number of distinct spectral regions. On the other hand, the number of raw data points is large compared to the number of individuals and the adjacent spectral points are strongly correlated. These characteristics suggest that a kernel parameterisation is a suitable choice to represent the complex but redundant data efficiently. A kernel is defined as an instance of the Gaussian density function (vector of probabilities) with a given centre and width. The parameterisation is formed by computing the dot product between one such instance and the vector $X_{(i)}$. This captures the local sum of intensities for a particular spectral region, denoted by $\phi_j(X_{(i)}, m_j, s_j)$, where m_j and s_j are the centre and width of the kernel j, respectively. In practise, the Gaussian function is truncated beyond three standard deviations since the contribution is negligible after that. Other kernel functions with wider tails could be used, but the resonance overlaps in the spectra suggest that little useful information can be extracted from the kernel tails.

After the non-linear parameterisation, the k kernel outputs ϕ are connected to the target variable y by a linear regression model, written as

$$\mu_i = w_0 \bar{X}_{(i)} + \sum_{j=1}^{k} w_j \phi_j(X_{(i)}, m_j, s_j),$$

which corresponds to assuming that the clinical measurement is related in an additive fashion to one or more metabolite resonances that can be detected by ^{1}H NMR of the biofluid. The term $w_0 \bar{X}_{(i)}$, where $\bar{X}_{(i)}$ is the mean intensity, is added

to include very wide biochemical effects, if any exist.

Obviously, the clinical measurements have errors from unspecified causes so a residual model is needed to handle the random noise. Outliers are common in this type of data sets and, evidently, a robust residual model is preferred, hence the Student's t-distribution with unknown degrees of freedom was chosen. The t-distribution can be represented by a mixture of Gaussians, defined as

$$\begin{aligned} y_i &\sim \mathrm{N}(\mu_i, V_i) \\ V_i &\sim \text{Inv-}\chi^2(\nu, \sigma^2), \end{aligned}$$

where V_i is the variance for the ith observation, and ν and σ^2 are parameters for the prior of V_i. To improve posterior sampling, the above can be further expanded to

$$\begin{aligned} y_i &\sim \mathrm{N}(\mu_i, \alpha^2 U_i) \\ U_i &\sim \text{Inv-}\chi^2(\nu, \tau^2), \end{aligned}$$

where α^2 is an additional parameter that allows jumps in common direction for all V_i.

After the model structure is established, the next important task is to define the prior distributions for the parameters. The mathematical formulation allows fast MCMC sampling from each conditional distribution in turn, and thus the particular sampler for each parameter is described. The likelihood parameters τ^2 and ν have the uninformative priors $p(\tau^2) \propto 1/\tau^2$ and $p(\nu) \propto 1/\nu$, respectively. In addition, the Gibbs' sampling steps for U_i, τ^2 and α^2 are defined in [1] page 305 and ν is sampled by slice sampling, see [2] for instance. The weights w in the

regression model have a Gaussian prior, written as

$$w_j \sim \mathrm{N}(0, \sigma_w).$$

The prediction results are not sensitive to the choice of the width σ_w of the weight prior, but the marginal posterior for the number of kernels can be affected, which is a well known problem for linear models (see [3] for example). Based on preliminary sensitivity analysis, $\sigma_w^2 = 10$ was selected. The conjugate Gaussian prior ensures that there is no need to explicitly sample these weights, instead they are integrated out analytically. This also reduces the dependencies in the joint posterior, greatly improving the speed of overall sampling. Equations for computing marginalized likelihood, and expectation and variance of μ_i can be obtained, for example, from [3].

It would be unjustified to prefer any spectral locations beforehand, so a uniform prior was chosen for the kernel centres, defined as

$$m_j \sim \mathrm{Unif}(1, m_{\mathrm{max}}).$$

Certain widths, on the other hand, can be preferred based on spectroscopic knowledge and practical arguments. Very small widths would be very unlikely from a molecular perspective and thus the informative Inv-χ^2 prior was chosen, written as

$$s_j \sim \text{Inv-}\chi^2(1, \sigma_s),$$

where $\sigma_s = 4$ (Hz). The kernel parameters m_j and s_j are sampled one at a time by slice sampling and, together with the model structure, this promotes quick mixing

with respect to the conditional distributions. The number of kernels should not be too strictly constrained, but slightly favoring smaller numbers will guard against useless kernels that might be included in a saturated linear regression. For this reason, a geometric prior was chosen, defined as

$$p(k) \propto 0.9^k.$$

Kernel number is sampled using a reversible jump MCMC algorithm from [4]. When adding a new kernel, the corresponding prior distributions are used as a proposal distributions for the kernel parameters.

The analysis software was implemented in the Matlab programming environment (The MathWorks Inc., Natick, Massachusetts, USA) with the MCMCStuff toolbox (http://www.lce.hut.fi/research/mm/mcmcstuff/).

References

1. Gelman A, Carlin JB, Stern HS, Rubin DR: *Bayesian Data Analysis.* Second edition, Chapman & Hall 2003

2. Neal RM: **Slice sampling.** *The Annals of Statistics* 2003, **31**:705-767

3. O'Hagan A and Forster J: *Kendalls's Advanced Theory of Statistics, Volume 2B, Bayesian Inference.* Second edition, Arnold 2004

4. Green P: **Reversible jump Markov chain Monte Carlo computation and Bayesian model determination.** *Biometrika* 1995, **82**:711-732
